# Evaluation of synergistic image registration for motion-corrected coronary NaF-PET-MR

**DOI:** 10.1098/rsta.2020.0202

**Published:** 2021-06-28

**Authors:** Johannes Mayer, Yining Jin, Thomas-Heinrich Wurster, Marcus R. Makowski, Christoph Kolbitsch

**Affiliations:** ^1^ Physikalisch-Technische Bundesanstalt (PTB), Braunschweig and Berlin, Germany; ^2^ Klinik für Kardiologie, Charité Campus Benjamin Franklin, Universitätsmedizin Berlin, Berlin, Germany; ^3^ Berlin Institute of Health, Berlin, Germany; ^4^ Department of Radiology, Charité, Universitätsmedizin Berlin, Berlin, Germany; ^5^ Department of Diagnostic and Interventional Radiology, Klinikum rechts der Isar, School of Medicine, Technical University of Munich, Munich, Germany

**Keywords:** synergistic image registration, cardiac motion, respiratory motion, motion-corrected image reconstruction, PET-MR, NaF-PET

## Abstract

Coronary artery disease (CAD) is caused by the formation of plaques in the coronary arteries and is one of the most common cardiovascular diseases. NaF-PET can be used to assess plaque composition, which could be important for therapy planning. One of the main challenges of NaF-PET is cardiac and respiratory motion which can strongly impair diagnostic accuracy. In this study, we investigated the use of a synergistic image registration approach which combined motion-resolved MR and PET data to estimate cardiac and respiratory motion. This motion estimation could then be used to improve the NaF-PET image quality. The approach was evaluated with numerical simulations and *in vivo* scans of patients suffering from CAD. In numerical simulations, it was shown, that combining MR and PET information can improve the accuracy of motion estimation by more than 15%. For the *in vivo* scans, the synergistic image registration led to an improvement in uptake visualization. This is the first study to assess the benefit of combining MR and NaF-PET for cardiac and respiratory motion estimation. Further patient evaluation is required to fully evaluate the potential of this approach.

This article is part of the theme issue ‘Synergistic tomographic image reconstruction: part 1’.

## 1. Introduction

Coronary artery disease (CAD) is one of the most common cardiovascular diseases. It can lead to heart failure, myocardial infarction and even sudden cardiac death. CAD is caused by a build-up of plaques in the coronary arteries which impair the blood supply of the heart. Previous studies have shown that the composition of these plaques could be an important diagnostic parameter to predict the progression of the disease and hence could be used to optimize patient treatment [1].

NaF-PET has been shown to be a promising clinical imaging modality to visualize vulnerable plaques, i.e. likely to rupture and to trigger a cardiac event [2,3]. Nevertheless, imaging the coronary arteries with PET is very challenging, because the heart is moving due to the heartbeat and respiratory motion during data acquisition. NaF uptake in the micro-calcification is very small and hence the physiological motion can lead to strong blurring of the uptake and make an accurate diagnosis challenging [4].

Simultaneous PET-MR has been proposed to improve the quality of cardiac PET by using respiratory and/or cardiac motion information estimated from MR images for motion correction [5–9]. Commonly, only MR information is used for motion estimation due to its excellent soft tissue contrast. Nevertheless, recent studies have shown that respiratory and cardiac motion estimation can be improved by combining motion-resolved MR and PET images for image registration for cardiac FDG-PET-MR [10]. Improved motion estimation then leads to better image quality of the final motion-corrected PET data.

So far, the advantages of synergistic image registration have only been shown for images with tracer uptake in the myocardium, i.e. a very large volume. In this paper, we evaluate if combining motion-resolved MR and NaF-PET image data also allows for a more accurate estimation of cardiac and respiratory motion of coronary plaques. The aim of this work is not to obtain the optimal image registration parameters but to investigate if synergistic NaF-PET-MR image registration is advantageous. This synergistic motion estimation approach is evaluated in simulated simultaneous NaF-PET-MR data and then applied to patients with calcified coronary plaques. The numerical simulations allowed for the evaluation of the accuracy of the obtained motion fields by comparing them to the ground truth motion information. In patients, the improvement of motion estimation and subsequent motion correction were evaluated visually.

## 2. Methods

**Figure 1.**
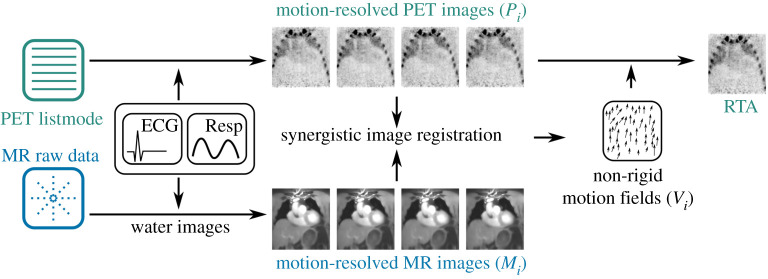
Overview of image registration and motion correction. (Online version in colour.)

The following gives an overview of PET and MR data acquisition and image reconstruction and the proposed synergistic motion estimation approach which was evaluated in this study. For more details especially of MR data acquisition, the reconstruction of different motion states and the estimation of attenuation correction (AC) maps, please refer to [11]. An overview of the methods is given in figure 1.

### (a) PET data acquisition and image reconstruction

PET data are acquired as listmode data. Cardiac motion states are obtained by separating the recorded events into different cardiac phases based on an external ECG device. Respiratory motion was monitored using a respiratory belt, which was recorded simultaneously to PET-MR acquisition. The amplitude of this motion signal is then used to separate the PET listmode data into different breathing states. In this study, we used four cardiac and four respiratory motion states, which has been shown to allow for accurate motion estimation in previous studies [12,13]. End-expiration and late-diastole were selected as reference motion phases. The separation of the data into different motion phases was carried out separately for respiratory and cardiac motion; joint binning into the cardio-respiratory motion phase was not done. For both cardiac and respiratory motion phases, hard binning was done. No rejection of irregular respiratory or cardiac cycles was carried out.

PET image reconstruction was carried out with an Order Subsets Expectation Maximization (OSEM) algorithm based on the open-source Synergistic Image Reconstruction Framework (SIRF) [14].

For AC, an AC map was used with only two tissue types: soft tissue and air outside the body. The AC map was calculated from the motion-corrected fat–water-separated MR data using intensity-based image segmentation to distinguish between the body and the outside air. For the body (including lung-tissue), an AC value of 0.1 cm^−1^ was used. The outside air was set to 0 cm^−1^ [15,16]. This ensured that the AC map does not lead to any motion artefacts (e.g. banana artefact at the dome of the liver) at the borders of different tissue type. These artefacts would otherwise impair the motion estimation for which an accurate uptake quantification is not required. Correction of scatter and random was also carried out [17,18].

### (b) MR data acquisition and image reconstruction

In order to be able to estimate cardiac and respiratory motion from the same MR raw data, we used a non-Cartesian three-dimensional acquisition scheme which has already been evaluated for various different motion estimation and motion correction applications [5,19]. Data acquisition is carried out continuously over multiple cardiac and respiratory cycles. A golden-angle-based increment of the non-Cartesian phase encoding points is used to ensure homogeneous filling of k-space over time and to allow for the retrospective reconstruction of arbitrary cardiac and respiratory motion states [20]. The MR data were separated into different motion states using the same signals as for PET acquisition. A total-variation-based iterative reconstruction scheme is applied [21]. Owing to the low signal-to-noise ratio (SNR) of the PET data, only four cardiac and four respiratory motion states were used in this study.

### (c) Synergistic motion estimation

The physiological motion between two motion states is estimated using image registration. In this study, we used a spline-based registration algorithm, which parametrizes the motion vector fields (*V_i_*) with spline functions. *V_i_* can then be determined by minimizing an image difference metric (D) between two two-dimensional or three-dimensional images (I):

2.1 $${\hat{V}}_{i}=\underset{V}{\min}D(I_{j},V\circ I_{i}).$$

In this case, *V_i_* describes the transformation from image *I_i_* to a reference image *I_j_*. The spline-parametrization already ensures smoothness of *V_i_*, which can be increased or decreased by changing the spline order (spline distance, sp) used to describe the motion. Further regularization (R), such as bending energy (be) penalty, can be added to this minimization to stabilize the solution in the presence of noise or undersampling artefacts:

2.2 $${\hat{V}}_{i}=\underset{V}{\min}[D(I_{j},V\circ I_{i})+\lambda R(V)].$$

Here, we use a synergistic approach for motion estimation, which combines both the motion-resolved MR (*M_i_*) and PET (*P_i_*) data in the minimization of (2.2):

2.3 $${\hat{V}}_{i}=\underset{V}{\min}[\left(1-w)D(M_{j},V\circ M_{i})+wD(P_{j},V\circ P_{i})+\lambda R(V\right)].$$

This formulation ensures that the obtained *V_i_* yields an optimal image metric value for both the MR and PET data. Especially for regions where there is no signal contrast (e.g. anatomical regions with constant PET uptake) in one modality but there is image information in the other, this approach is expected to yield superior motion information. The parameter *w* is a weighting factor which can be used to choose how much each modality contributes to the registration, e.g. for *w* = 1 only the PET data are used, for *w* = 0 only the MR data are used and for *w* = 0.5 both modalities contribute equally to the solution of the minimization.

### (d) Motion correction

The obtained *V_i_* can then be used to transform all different motion states to the reference motion state in order to compensate for motion. The transformed images can then be combined into a single motion-corrected image. This so-called reconstruct–transform–average (RTA) approach therefore is an image-based averaging of the reconstructed PET data after motion correction, and it is very fast, because it does not involve any additional image reconstruction. Improved image quality could be achieved using an motion-compensated image reconstruction approach, where *V_i_* is directly used at each iteration of the image reconstruction to correct for motion. Nevertheless, this is computationally much more demanding than the RTA method. In this study, using RTA allowed us to compare many different registration parameter settings efficiently. Also, our aim here was to compare different image registrations to each other rather than obtaining the best image quality.

## 3. Experiments

### (a) Data acquisition

Simultaneous PET and MR data were acquired in patients with suspected CAD on a mMR Biograph 3T scanner (Siemens Healthineers, Erlangen, Germany). From this patient group, we selected four patients (all male, 77.50 ± 7.14 kg, 66.25 ± 6.7 years) with uptake in coronary plaques which was also visible in the motion-resolved PET images. PET data were acquired for 55.42 ± 29.34 min and the MR data (FOV: 288 mm^3^, 1.5 mm^3^ image resolution, multi-echo (TE: 2.6/4.1/5.6 ms) gradient-echo sequence, FA: 15°, TR: 7.6 ms) for 12 : 25 min during part of the PET acquisition. The k-space was oversampled by a factor 2. Before the MR scan, a T1 contrast agent (Gadovist) was injected to the patient as part of the clinical protocol. The administered PET dose was 167.25 ± 15.54 MBq and the PET scan started about 2 h after tracer injection. Data from the entire PET scan were used for motion correction to ensure the maximum available tracer uptake in the plaques. During the PET scan, several clinical MR scans were acquired and some of them required respiratory gating and cardiac triggering. Therefore, the total PET duration varied between patients and also the time, when the MR scan used for motion correction was acquired, varied between patients. Nevertheless, the MR scan was always scheduled to take place towards the end of the PET scan. Respirator belt signal and RR duration estimated from the external ECG are shown in electronic supplementary material, figure S1.

The MR data were transformed into the PET coordinate system and resampled to the PET resolution. Because the MR data were acquired in a sagittal orientation without any angulation, interpolation was only required in order to resample from the MR resolution (1.5 × 1.5 × 1.5 mm^3^) to the PET resolution (2.1 × 2.1 × 2.0 mm^3^).

### (b) Data simulation

In order to evaluate the accuracy of the estimated *V_i_*, a numerical simulation framework was used to simulate a simultaneous PET and MR data acquisition during free-breathing and over multiple cardiac cycles [22]. The phantom underlying the simulation was voxelized anatomy based on the XCAT phantom using an isotropic FOV of 288 mm at 1.5 mm resolution with dense motion vector field on the same grid. The simulated geometry was from a Siemens Biograph mMR scanner using an analytic forward projection of a known tracer distribution with subsequent addition of Poisson noise. A simulation of scatter and random, detector normalization and point-spread function effects were omitted.

For MR, realistic longitudinal relaxation times were assigned to different tissue types, and a signal model for the above described MR acquisition was applied to simulate the MR signal behaviour. Complex Gaussian noise was added to achieve SNR comparable to *in vivo* acquisitions. All the scan parameters such as image resolution, FOV and k-space trajectory were kept the same as for the actual *in vivo* scan. In addition, a PET listmode scan was simulated with uptake in a plaque in the right coronary artery.

The maximum amplitude was 10.3 pixel (15.5 mm) and 5.2 pixel (7.8 mm) for respiratory and cardiac motion, respectively. Motion vector fields for respiratory and cardiac motion are shown in electronic supplementary material, figure S2. In order to simulate residual motion within each reconstructed motion phase, we simulated 12 cardiac and 12 respiratory phases. From these data, four cardiac and four respiratory phases were reconstructed. For the simulations, either cardiac or respiratory motion was simulated but not both in order to be able to assess both types of motion separately. The respiratory belt and ECG signal of one of the patients were used as respiratory and cardiac surrogate signals to map the motion vector fields to the MR and PET raw data.

**Figure 2.**
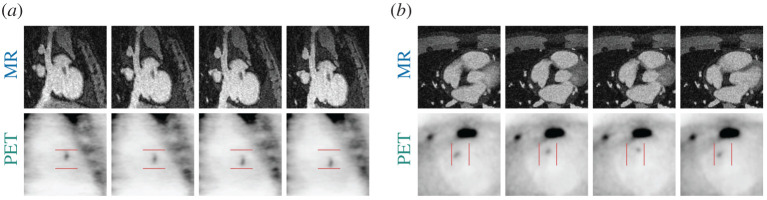
Simulated respiratory (*a*) and cardiac (*b*) motion-resolved MR and PET (TBR 3) images. Location of plaque is highlighted by arrows in MR and PET data. (Online version in colour.)

The simulated activity in the plaque was varied in order to assess how different uptake strength influences the contribution of the PET data to the motion estimation. It was set such that the target-to-background ratio (TBR) for the motion-corrected case was 29.4, 7.7, 2.7 and 1.4. For the case with uncorrected respiratory motion, TBR was 14, 3.9, 1.7 and 1.1. For easier readability, we refer to these differently simulated datasets as TBR 30, TBR 8, TBR 3 and TBR 1.4. The uptake was varied and figure 2 shows the simulated motion-resolved MR and PET images for TBR 3.

The simulated data are available here: https://oar.ptb.de/resources/show/10.7795/710.20201113.

### (c) Motion estimation

Motion estimation was carried out with a spline-based image registration tool (Medical Image Registration ToolKit (MIRTK), [23]). As mentioned above image registration optimizes an image similarity metric while the computed transformations are subject to further regularization terms. The final registration accuracy strongly depends on the registration parameters and in order to avoid any bias of the results due to the choice of these parameters, we evaluated each registration for a range of parameters. In this study, we restricted these parameters to the bending energy term for the regularization and the spline distance used for the parametrization of the motion fields to keep the size of the parameter space reasonable. The be (corresponding to *λ* in equation (2.3)) was chosen from [0, 3 × 10^−4^, 6 × 10^−4^, 8 × 10^−4^, 1.1 × 10^−3^, 2.2 × 10^−3^, 3.3 × 10^−3^, 4.4 × 10^−3^, 5.5 × 10^−3^, 6.7 × 10^−3^, 7.8 × 10^−3^, 8.9 × 10^−3^, 1 × 10^−2^] and the spline distance (sp) was chosen from [4,9,14,19,24]. In addition, the weight between PET and MR data was chosen from [0, 0.25, 0.5, 0.75 and 1.0]. This led to 325 registrations for each motion state. Image registration was carried out in a small region of interest (ROI) (approx. 50^3^ voxels) around the plaque in order to speed up image registration. MR and PET image intensities were both normalized prior to image registration in order to ensure they contribute similarly to the cost function in equation (2.3). Normalized mutual information was used as a similarity metric (*D* in equation (2.3)).

### (d) Evaluation of motion estimation

The numerical simulation does not just provide the MR and PET raw data, but it also yields ground truth vector fields ($V_{i}^{GT}$) describing the simulated respiratory and cardiac motion. The accuracy of motion estimation was then evaluated by calculating the root mean squared error ($V_{i}^{\mathrm{err}}$) between *V_i_* obtained with a certain parameter combination (sp, be, *w*) and $V_{i}^{GT}$:

$$V_{i}^{\text{err}}(sp,be,w)=\frac{1}{3N_{ROI}}\sqrt{\underset{N_{ROI}}{\sum }\underset{x,y,z}{\sum }{\left(V_{i}^{GT}-V_{i}^{\;}(sp,be,w)\right)}^{2}}.$$

The error was calculated for each motion state in a ROI around the simulated plaque. In order to assess the quality of the image registration, the maximum error over all motion states was then used (*V*^err^). In addition to *V*^err^, we also estimated the difference between the motion field amplitudes (*V*^amp^) neglecting the orientation of the motion vectors and the difference between the orientation of motion fields (i.e. the angle between the vectors, *V*^ang^) neglecting the amplitude of the vectors.

For the final assessment, we calculated the minimum error over all (sp, be) combinations. This corresponds to the motion estimation with the highest accuracy. Nevertheless, for *in vivo* applications, the best parameter combination of sp and be is often not known, and, hence, we also calculated the median value of *V*^err^ as an indication of the robustness of the motion estimation.

In addition to *V*^err^, we also compared a line profile through plaque uptake for the RTA images to the uncorrected images and gated images. For the gating, we selected the reference motion state of the image registration. In order to make the line profile more robust, we calculated it along a width of three voxels and averaged these three profiles.

## 4. Results

**Figure 3.**
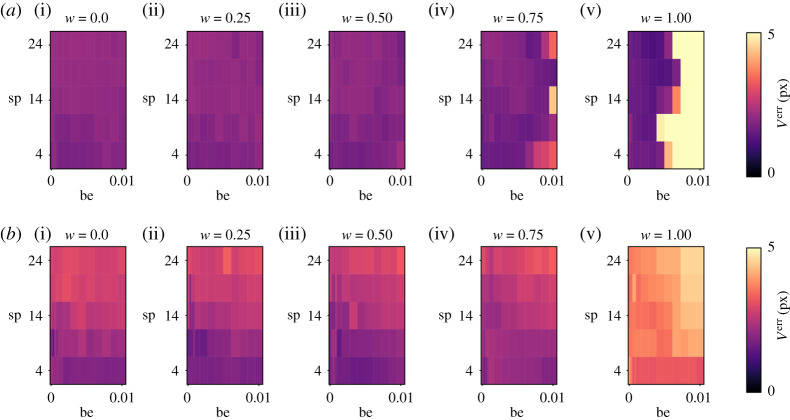
Motion vector field error (*V*^err^) calculated for different bending energy (be), spline distance (sp) and different *w* for (*a*) respiratory motion estimation and (*b*) cardiac motion estimation. The PET images used for the image registration were TBR 3. (Online version in colour.)

**Figure 4.**
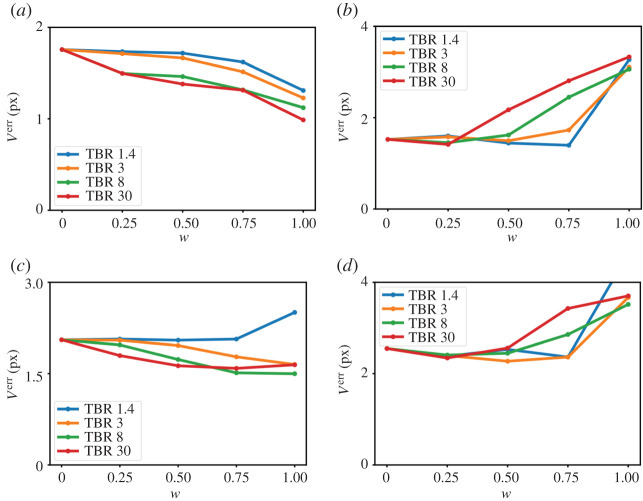
Error of motion estimation (*V*^err^) for different weighting factors (*w*) und plaques with different TBR for (*a*,*c*) respiratory and (*b*,*d*) cardiac motion estimation. (*a*,*b*) Minimum and (*c*,*d*) median of *V*^err^ over all regularization parameters. (Online version in colour.)

*V*^err^ is shown in figure 3 for different regularization parameters (be, sp) and different weighting factors *w* for TBR 3. Combining both PET and MR image data improves the accuracy of the image registration leading to smaller values of *V*^err^. Exemplary motion fields are shown in electronic supplementary material, figures S3–S6. The minimum and median value of *V*^err^ for each *w* is displayed in figure 4 for the different simulated TBR values. For respiratory motion, *w* = 0.75 shows the best accuracy and robustness. Using only PET data (*w* = 1) yields even better results, but only if TBR is high enough and fails for TBR 1.4. For cardiac motion estimation, high TBR values can reduce the accuracy of motion estimation. Nevertheless, for TBR ratios expected in *in vivo* scans (TBR 1.4 and 3), the synergistic registration leads to an improvement of the accuracy of image registration. Averaged over all four TBR values, using both MR and PET data reduces the minimum of *V*^err^ by 18.0 ± 7.5% for respiratory motion (*w* = 0.75) and by 0.86 ± 0.52% for cardiac motion (*w* = 0.25) compared to using only MR. The median of *V*^err^ is reduced by 15.5 ± 10.4% (*w* = 0.5) and by 6.6 ± 0.9% (*w* = 0.25) for cardiac motion. Electronic supplementary material, figures S6 and S7 show *V*^amp^ and *V*^ang^.

**Figure 5.**
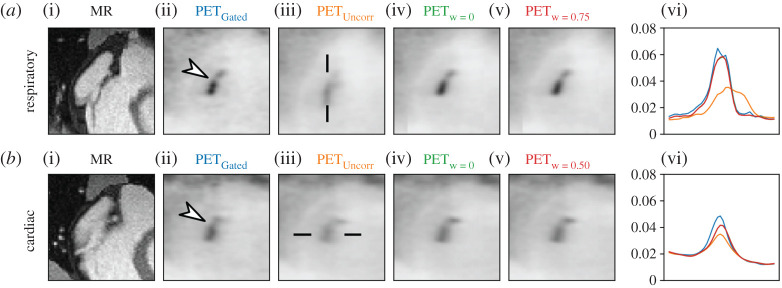
Results of numerical simulation (TBR 3). Respiratory and cardiac motion-corrected PET images with different weights compared to the uncorrected (PET_uncorr_) and gated (PET_Gated_) PET (TBR 3) images. Line plots through the plaque uptake at a location indicated by black lines in PET_uncorr_. Motion-corrected MR images are also shown as anatomical references. Location of plaque is highlighted by arrows in MR and PET data. (Online version in colour.)

The RTA images for different registration parameters are shown in figure 5 for TBR 3. For *w* = 0 and *w* > 0 the RTA images show improved visualization of the plaque uptake and line profiles which are closer to the gated PET data compared to the uncorrected PET data. The motion fields used here for RTA were selected based on the median of *V*^err^. Although for TBR 3 *w* = 0.75 and *w* = 0.50 lead to a reduction of *V*^err^ of 13.6% and 10.9% compared to *w* = 0, for respiratory and cardiac motion estimation, respectively, the effect on the final RTA images is hardly noticeable.

**Figure 6.**
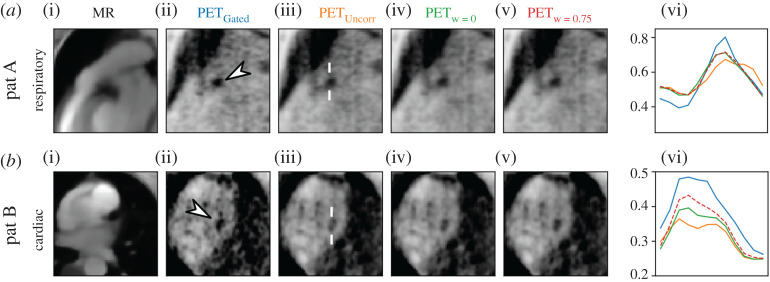
Results of patient data. Respiratory and cardiac motion-corrected PET images with different weights compared to the uncorrected (PET_uncorr_) and gated (PET_Gated_) PET images. Line plots through the plaque uptake at a location indicated by white lines in PET_uncorr_. Motion-corrected MR images are also shown as anatomical references. (Online version in colour.)

Figure 6 shows the results of respiratory and cardiac motion correction for two different patients. For patient A, both the MR-only and joint PET-MR image registration improve the visualization of the uptake in a similar way. For patient B, the joint PET-MR image registration leads to a plaque visualization which is closer to the gated PET image than the MR-only registration, suggesting that the combination of PET and MR improves the motion estimation. The motion fields were selected with sp = 9 and be = 1.1 × 10^−3^.

## 5. Discussion

We have shown in numerical simulations that combining both PET and MR data in a synergistic image registration can improve the accuracy of the motion fields. In patients, we have shown that synergistic image registration can improve the final image quality.

The main limitation of this approach is that the uptake in plaques has to be high enough, such that it is visible in each gated PET image. The image registration evaluates the image similarity between different gated images and hence if the PET uptake in plaque for each gate is below the noise level in the image, accurate motion information cannot be obtained. This also limits the number of respiratory and cardiac motion states which can be used for motion estimation. In order to overcome this problem, motion estimation and image reconstruction would need to be combined into an optimization scheme, such that the motion estimation is evaluated on the motion-corrected image data rather than on each motion gate [24,25]. Owing to the small size of the coronary plaques, partial volume effects strongly affect the measured uptake. Previous studies have shown that combining motion correction and partial volume correction can further improve the quantification of small uptake regions [12,26].

For the numerical simulation, cardiac and respiratory motion were simulated separately. Owing to the low uptake in the *in vivo* data, it was not possible to split the PET data into 4 × 4 cardio-respiratory motion states, but each type of motion estimation had to be carried out with the other motion type still present, potentially impairing image registration. In practice, a three-step approach could be used. First respiratory motion is estimated, and then cardiac gates are reconstructed with respiratory motion correction. In the last step, the cardiac and respiratory motion vector fields are combined into a cardio-respiratory motion model and applied for the final image reconstruction [5,6,9].

In this study, we only used four cardiac and four respiratory motion states due to low uptake values in the *in vivo* data, which led to residual motion in each gate. Nevertheless, previous studies using PET/CT to image NaF uptake in coronary plaques have shown that uptake in coronary plaques was visible (and could be used for motion estimation) even after splitting a 20 min PET scan into 10 cardiac phases [13]. Therefore, we believe that an optimized PET-MR acquisition could reduce the required PET scan time and allow for more motion states which would improve motion estimation.

In this study, we used a standard respiratory belt signal to separate the MR and PET data into respiratory motion states. This signal is pre-processed by the PET-MR scanner, which can lead to clipping of values outside of a certain range. Using external respiratory monitoring devices independent of the PET-MR scanner could overcome this issue.

Figure 4 shows that adding PET information to the image registration decreases the accuracy of cardiac motion estimation for very high TBR and *w* > 0.25. This shows that for such high TBR, the image registration optimizes the image quality metric for the plaque but this does not necessarily correspond to the true underlying motion. For lower TBR, the MR information ensures that the motion estimation follows the true underlying motion. This is also confirmed by the motion vector fields for TBR 3 and TBR 30 shown in electronic supplementary material, figures S3–S6. For respiratory motion, both TBR values lead to motion vector fields which follow the ground truth vector fields well. For cardiac motion, image registration leads to inaccurate motion estimation for TBR 30. Although this leads to a high signal in the plaque (which can be seen in the RTA images), it does not describe the true plaque uptake. This also highlights that the accuracy of motion correction (especially for complex motion such as cardiac motion) can only be fully assessed if ground truth motion vector fields are available or if the true tracer distribution is known.

For *in vivo* applications, such high tracer concentrations are not to be expected and hence this inaccurate motion estimation due to high TBR is less of a problem. Although our results suggest that *w* should be optimized for respiratory and cardiac motion correction separately, a compromise for *w* would be more practical.

For *in vivo* applications, we verified the image registrations by comparing profiles of the plaque uptake to a gated PET image. We did not use any quantitative metrics such as TBR, because, as discussed above, an increase in the PET uptake value does not necessarily correspond to more accurate motion estimation. In addition, this is only a first explorative study to assess the potential benefits of synergistic motion estimation for cardiac NaF-PET-MR and future studies with higher numbers of patients are required.

The MR image quality of the motion-resolved data was very high because a T1 contrast agent was used which improved the contrast-to-noise ratio between blood and myocardium. Also, an advanced total-variation-regularized image reconstruction was used. Therefore, the MR only (*w* = 0) motion estimation is already of high quality and adding further dynamic PET information only leads to a visible improvement in very few cases (figure 6). For PET, only a standard OSEM reconstruction was used without any regularization. Future work will focus on a more advanced PET reconstruction to improve the quality of the dynamic PET data in order to increase the positive effect of the synergistic image registration on the final motion-corrected images.

## 6. Conclusion

In this study, we analysed the effect of a synergistic respiratory and cardiac motion estimation for NaF-PET-MR imaging in numerical simulations and *in vivo* data obtained in CAD patients. Combining both MR and PET data can improve the accuracy and also robustness of the image registration. Nevertheless, this effect strongly depends on the uptake in the plaque and how well the underlying motion is resolved. Both for numerical simulation and *in vivo* data the quality of the MR images was very high and hence the positive effect of using PET information for the image registration was very small. This could be improved with advanced PET image reconstruction or by using a joint motion estimation and image reconstruction approach [24,25].

## Data accessibility

The simulated respiratory and cardiac resolved MR data and the corresponding PET data for different TBR are available on: https://oar.ptb.de/resources/show/10.7795/710.20201113.
